# The structural basis of unique substrate recognition by Plasmodium thymidylate kinase: Molecular dynamics simulation and inhibitory studies

**DOI:** 10.1371/journal.pone.0212065

**Published:** 2019-02-07

**Authors:** Mahmoud Kandeel, Yukio Kitade, Abdulla Al-Taher, Mohammed Al-Nazawi

**Affiliations:** 1 Department of Physiology, Biochemistry and Pharmacology, Faculty of Veterinary Medicine, King Faisal University, Hofuf, Alahsa, Saudi Arabia; 2 Department of Pharmacology, Faculty of Veterinary Medicine, Kafrelsheikh University, Kafrelsheikh, Kafrelsheikh, Egypt; 3 Department of Applied Chemistry, Faculty of Engineering, Aichi Institute of Technology, Yachigusa, Yakuza, Toyota, Japan; 4 Department of Chemistry and Biomolecular Science, Faculty of Engineering, Gifu University, Yanagido, Gifu, Japan; Universidade Nova de Lisboa Instituto de Tecnologia Quimica e Biologica, PORTUGAL

## Abstract

*Plasmodium falciparum* thymidylate kinase (PfTMK) showed structural and catalytic distinctions from the host enzyme rendering it a hopeful antiprotozoal drug target. Despite the comprehensive enzymologic, structural, inhibitory and chemical synthesis approaches targeting this enzyme, the elucidation of the exact mechanism underlying the recognition of the atypical purine substrates remains to be determined. In this study, molecular dynamics (MD) simulation of a broad range of substrates and inhibitors as well as the inhibitory properties of deoxyguanosine (dG) derivatives were used to assess the PfTMK substructure molecular rearrangements. The estimated changes during the favourable binding of high affinity substrate (TMP) include lower interaction with P-loop, free residue fluctuations of the lid domain and the average RMSD value. The RMSD of TMP complex was higher and more rapidly stabilized than the dGMP complex. The lid domain flexibility is severely affected by dGMP and β-thymidine derivatives, while being partially fluctuating with other thymidine derivatives. The TMK-purine (dGMP) complex was slowly and gradually stabilized with lower over all structure flexibility and residue fluctuations especially at the lid domain, which closes the active site during its catalytic state. Thymidine derivatives allow structure flexibility of the lid domain being highly fluctuating in α- and β-thymidine derivatives and TMP. dG derivatives remains less efficient than thymidine derivatives in inhibiting TMK. The variations in the structural dynamics of the P-loop and lid domain in response to TMP or dGMP might favour thymidine-based compounds. The provided MD simulation strategy can be used for predicating structural changes in PfTMK during lead optimization.

## Introduction

During the search for new drug targets against world health hazardous protozoal diseases, we identified PfTMK as a new promising drug target [1]. Mutational, biochemical and biophysical approaches revealed broad spectrum substrate binding efficiency of PfTMK [2]. PfTMK is a pyrimidine metabolizing enzyme; unexpectedly, it was able to bind the guanylate, deoxyguanylate and inosinylate compounds, which are purine derivatives [3, 4]. This unique feature was proposed as a starting point for finding protozoal specific inhibitors since the human thymidylate kinase (hTMK) is a very specific pyrimidine only binding enzyme.

The structure basis of substrates recognition by PfTMK by using X-ray crystallography revealed significant structure rearrangements in PfTMK that ensures wider substrate spectrum and faster metabolism of AZT (3'-azido-3'-deoxythymidine)-MP (monophosphate), which is a feature of prokaryotic TMKs [5].

Based on the provided unique biochemical and structural features, several scaffolds of inhibitors were designed and tested against PfTMK. At first, 2',3' dideoxycarbocyclic derivative of thymidine showed strong PfTMK inhibition in the low micromolar range [6]. Additionally, the fluorinated dideoxy derivative (-)-7 exhibited improved inhibition efficiency [7]. Several 5'-urea-α- and β-thymidine derivatives were synthesized and showed moderate inhibitory potency against PfTMK [8]. QSAR, pharmacophore mapping and docking studies for α- and β-thymidine analogs binding with PfTMPK revealed the importance of–NH fragment and urea derivative of thymidine in the inhibition of PfTMK [9]. More recently, a trial was made to improve the moderate PfTMK inhibitory effect of α-thymidine derivatives. N-(5'-deoxy-α-thymidin-5'-yl)-N'-(4-(2-chlorobenzyloxy)phenyl)urea was used as a parent compound owing to its effective inhibition of *P*. *falciparum* growth. However, the new derivatives were only effective in the micromolar range [10].

Despite the application of various biochemical, structural and chemical synthesis techniques in PfTMK inhibition, the exact molecular mechanisms underlying the recognition of inhibitors, especially the guanosine and α-thymidine inhibitors, is still not well understood. Resolving the molecular changes during each substrate interaction with PfTMK will be important in optimizing new inhibitors. In order to perform this task, we used the molecular dynamics approach. MD simulation would resolve the facts hidden within PfTMK and reveal the substructure responses to different inhibitors. Understanding such mechanism is expected to help in the design of stronger PfTMK inhibitors. Most PfTMK inhibitory studies were using thymidine derivatives. Due to lack of inhibitor data by using dG derivatives, several compounds were analysed by inhibitory assays, docking studies and ligand-protein interactions. Overall, thymidine and deoxyguanosine derivatives interactions with PfTMK were evaluated.

## Materials and methods

### PfTMK structures preparation

The structures of PfTMK bound with different compounds were retrieved from the protein data bank (PDB). The PDB IDs and their ligands contents are presented in Table 1. The retrieved structures were prepared by correction for missing atoms, bonds or side chains. During MD simulation, two replicates of structures were used, either monomer or dimers of each PDB structure file. In every structure file, monomer no. B is removed followed by energy minimization.

**Table 1:** The protein data bank IDs and ligand contents in PfTMK structures.

PDB ID	ligand	abbreviation
**2WWF**	TMP and ADP	TMK-TMP
**2WWG**	dGMP and ADP	TMK-dGMP
**2WWH**	Ap5dT	TMK-Ap5dT
**2WWI**	AZTMP and ADP	TMK-AZTMP
**2YOF**	(thio)urea- β-deoxythymidine inhibitor	TMK-TUBdT
**2YOG**	(thio)urea- α-deoxythymidine inhibitor	TMK-TUAdT

### Molecular dynamics simulation

YASARA Structure software (version 14.12.2) was used for all MD simulations by opting for the use of AMBER14 as a force field. The simulation cell was allowed to include 20 Å surrounding the protein and filled with water at a density of 0.997 g/ml. Initial energy minimization was carried out under relaxed constraints using steepest descent minimization. Simulations were performed in water at constant pressure with temperature at 298 K. In order to mimic physiological conditions, counter ions were added to neutralize the system; Na or Cl was added in replacement of water to give a total NaCl concentration of 0.9%. pH was maintained at 7.4. The simulation was run at a constant pressure and temperature (NPT ensemble) for 50 ns. All simulation steps were run by a preinstalled macro (md_runfast.mcr) within the YASARA package. Data were collected every 250 ps. MD simulation results were estimated for both monomers and dimers of PfTMK. The enzyme naturally exists as dimer. Momoners were obtained by deletion of one unit from the obtained structure file.

### Calculation of secondary structure content

The secondary structure contents of PfTMKs were analysed before and after MD simulation using the secondary structure content estimation implemented in the YASARA software. The results comprise the percentage of contribution of helix, sheet, turn, or coil to the total structure composition.

### Expression and purification of PfTMK

Expression and purification were carried out as previously described [1, 4]. Briefly, ampicillin treated LB medium was used to grow recombinant *E*. *coli* overnight. The primary culture was used to infuse 2 liters of LB medium. Growth of *E*. *coli* was continued for 4 h before the addition of IPTG (isopropyl β-D-1-thiogalactopyranoside) to a final concentration of 1 mM. Expression was induced and LB medium was incubated for 4h following collection of cells by centrifugation at 5000 rpm for 15 min. Extraction and purification steps were implemented in 25 mM Tris-HCl buffer pH 7.2 containing 150 mM NaCl. Purification of PfTMK was by two affinity and gel filtration steps.

### Thymidylate kinase inhibitor assay

The TMP kinase activity was measured spectrophotometrically using an enzyme-coupling assay. The assay is based on the decrease in absorbance at 335 nm due to conversion of NADH to NAD. The activity solution contained essential activity elements as KCL, MgCl2 as well as coupling enzymes 2U pyruvate kinase, 2U lactate dehydrogenase and their forerunner phosphoenolpyruvate [1, 2]. For assessment of inhibitors, the initial reaction rates were measured in the presence and absence of inhibitors as previously described [2, 6, 7].

### Docking of dG derivatives

Several dG compounds (Table 2) were prepared by Chem3D software for the Molegro 5.5 docking module. The compounds were 3D optimized and saved as SD files. At first, the docking template was prepared by Molegro 5.5 software, based on the coordinates of dGMP binding of PDB ID 2WWG. Docking was then run using the default parameters in the Molegro docking wizard.

**Table 2:** dG derivatives used in docking and inhibitory studies.

Compound name	Docking score	Interaction energy	Ki (μM)
**deoxyguanosine**	-133	-145	270
**8-Hydroxy-2'-deoxyguanosine**	-137	-144	180
**Guadecitabine**	-220	-242	90
**Guanosine diphosphate mannose**	-199	-227	>1000

## Results

### Stability of trajectories during MD simulation

The mean RMSD value for PfTMK structures (Table 3) showed that TMK-dGMP was the lowest (0.75 Å), while TMK-AZTMP and TMK-TUBdT were the highest (1.28 Å). The mean value of receptor RMSD during MD is the lowest in TMK-TUAdT, which indicating the lowest conformational change and better ligand interaction with PfTMK. This value is greatly improved over TMK-TUBdT, which indicates that there would be more stable interactions of α-thymidine over β-thymidine derivatives. The RMSD of TMK-AZTMP is the highest among all complexes, indicating lower interaction efficiency of PfTMK with AZTMP.

**Table 3:** Mean backbone and receptor RMSD.

TMK complex	Mean backbone RMSD (Å)	Receptor RMSD (Å)
TMK-TMP	0.88	0.77
TMK-dGMP	0.75	0.68
TMK-Ap5dT	1.13	1.15
TMK-AZTMP	1.28	1.32
TMK-TUBdT	1.28	0.82
TMK-TUAdT	1.02	0.67
ApoTMK	1.2	—

**Fig 1:**
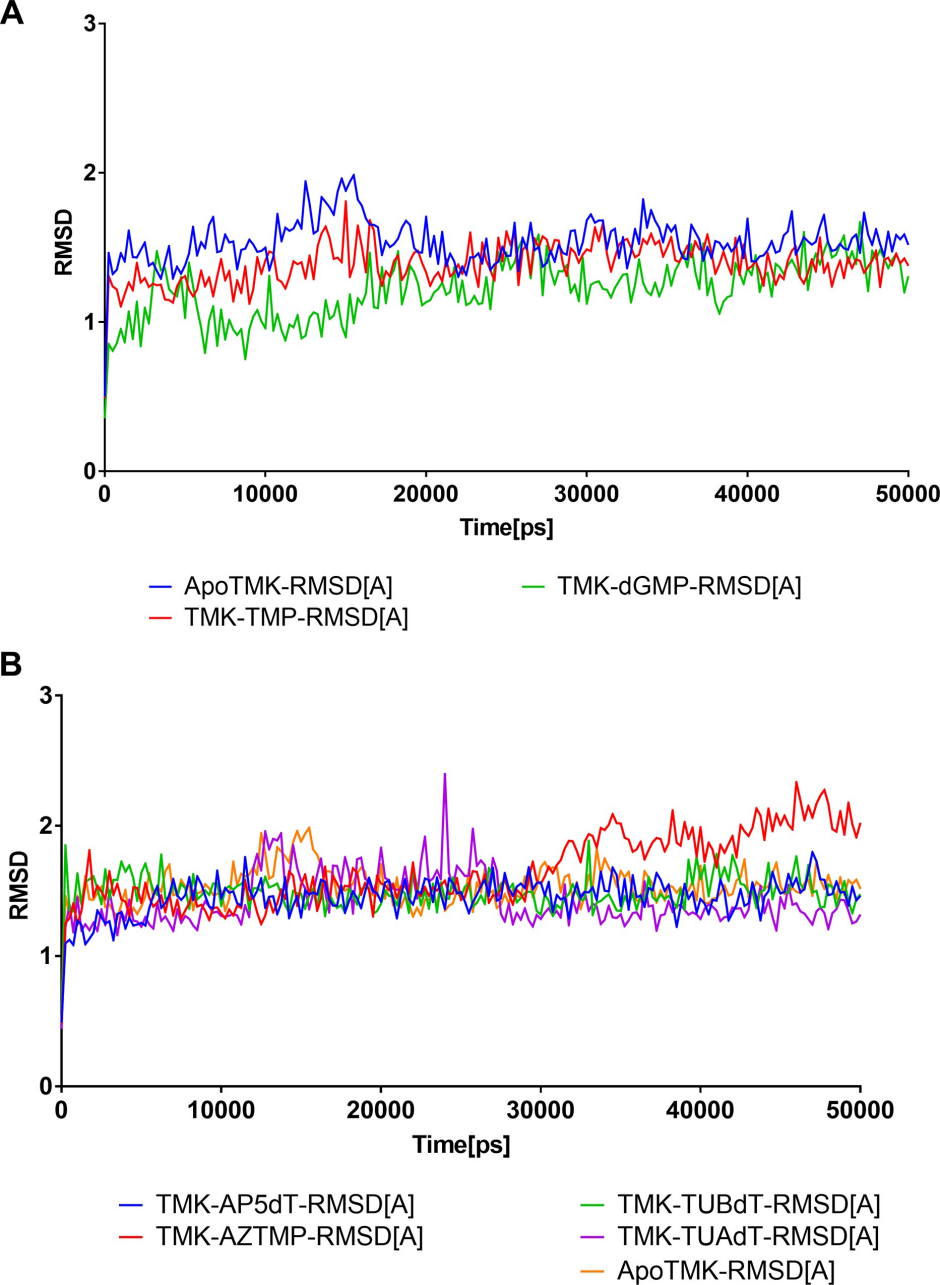
RMSD of PfTMK complexes. The RMSD (Å) is plotted over the simulation time in ns. The time (X axis) is represented in ps units.

Fig 1 shows the backbone alpha carbon RMSD of PfTMK complexes. In Fig 1A, TMK-TMP and TMK-dGMP were plotted with ApoTMK. All structures reached stability within 17 ns and remained stable throughout the rest of the simulation. Tracing changes in RMSD throughout the MD simulation time highlights the general strength of both TMP and dGMP in producing lower RMSD and lower conformational changes, compared with ApoTMK (blue line). While dGMP demonstrated lower RMSD during most of the simulation time, the RMSD plot was almost similar to TMK at the end of the simulation (Fig 1A). A5PdT showed a very stable profile starting from the first few ns at the beginning of the simulation (Fig 1B). By contrasting the α- and β-thymidine derivatives RMSD profile, it would be clear that β-thymidine derivatives retained a stable profile throughout the simulation time, while the alpha derivative showed abrupt thrills of RMSD fluctuations in the middle of simulation time (Fig 1B). Surprisingly, AZTMP, which is a unique substrate for PfTMK, showed abrupt drift and instability at 28 ns and continued to the end of simulation time.

### Structure flexibility analysis

**Fig 2:**
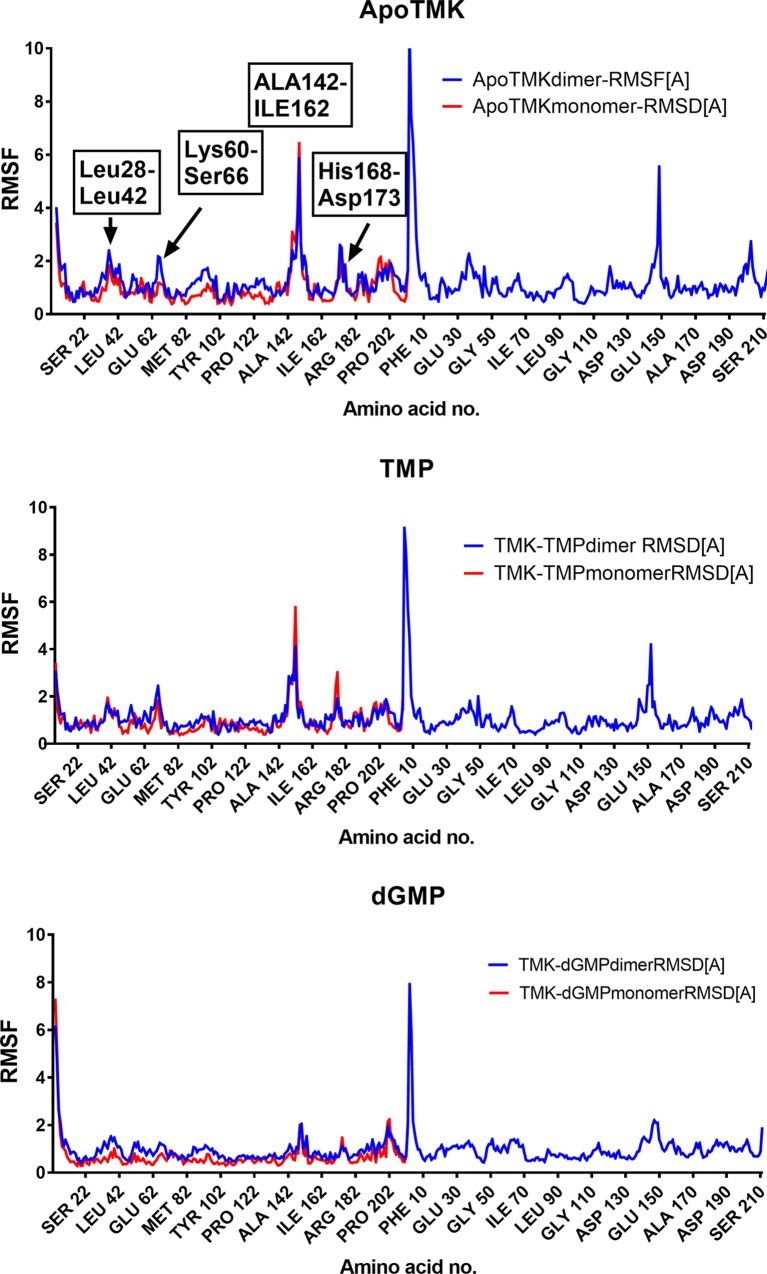
RMSF of all residues in ApoTMK or bound with TMP or dGMP.

**Fig 3:**
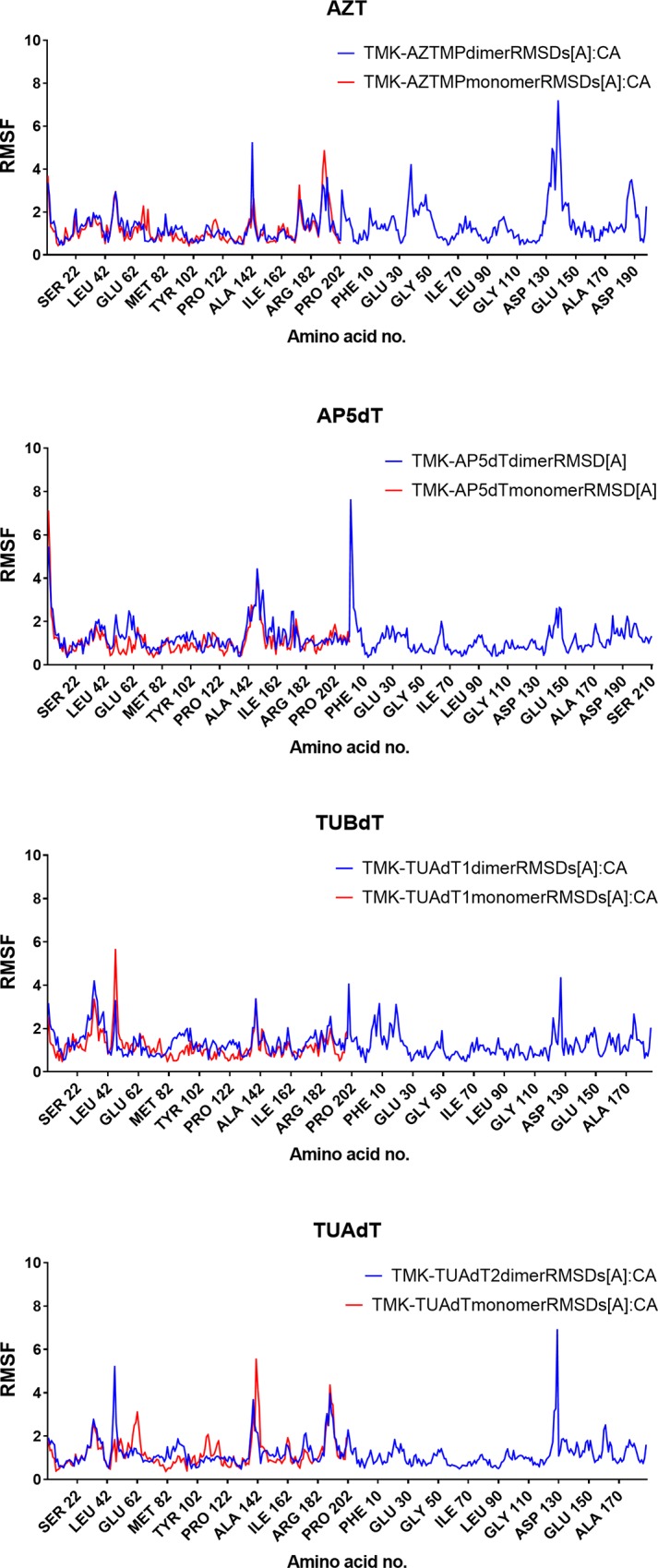
RMSF of all residues in ApoTMK or TMK bound with AZTMP, Ap5dT, TUBdT and TUAdT.

PfTMK backbone flexibility analysis during MD simulation was evaluated by plotting RMSF of all residues of PfTMK against simulation time (Figs 2 and 3). Fig 2 shows the comparison of RMSF values in Apo-TMK compared with the natural substrates TMP and dGMP. RMSF showed conserved and different values among aligned PfTMK complexes. With respect to the RMSF changes, there are four observed regions with noticeable RMSF changes. The greater RMSF changes were in the range between ALA142 to ILE162 with RMSF value up to 6 Å. Additionally, there are three more regions with minor RMSF changes including Leu28-Leu42, Lys60-Ser66 and His168-Asp173. The most important changes were observed in ALA142-ILE162, which are located in the lid domain. The magnitude of fluctuations was the highest in ApoTMK, followed by TMP and lastly dGMP. This implies that there are lower flexibility of PfTMK side chains, especially the lid domain, during the binding of dGMP.

A surprising finding is that AZT shows asymmetric changes across the two monomer of PfTMK dimer (Fig 3). The lid domain and N-terminal region showed higher RMSD values. Compared with ApoTMK and TMP-TMK, TUBdT showed lower RMSD of lid domain residues (<4 Å) and comparatively high RMSDs at the N-terminal region. Peaks of high RMSDs at the N-terminal region were observed with Leu32-ASN34 (~4.1Å) and Arg47 (5.8 Å). TUAdT displayed a profile similar to TUBdT in its N-terminal region (Fig 3). However, the fluctuations in lid domain residues were higher in TUBdT. Therefore, the amplitude of lid domain movement was in the following order ApoTMK> TMP> AZT> TUBdT> Ap5dT> TUAdT> dGMP. Therefore, the lid domain undergoes little structural arrangements in the presence of guanylate substrate compared with thymidylate.

### Secondary structure content

The secondary structure content of PfTMK is composed of a combination of helix, sheet, trun and coil with a varying contribution. In all PfTMK structures bound with compounds, there is a 2–5 fold increase in turn content compared with ApoTMK with a corresponding decrease in helical and coil % (Table 4). TMK-dGMP showed the highest helix % and lowest sheet and coil content.

**Table 4:** Secondary structure contents (%) of PfTMK bound with different compounds.

	ApoTMK	TMK-TMP	TMK-dGMP	TMK-Ap5dT	TMK-AZTMP	TMK-TUBdT	TMK-TUAdT
**helix**	45.8	41.3	45.7	41.4	44	45.2	42.2
**sheet**	19.2	19.2	15.2	18.1	18	19.3	20.1
**turn**	2.8	9.6	11.4	11.4	8	6.1	10.1
**coil**	33.2	29.8	25.7	29	30	29.4	25.6
**3–10 helix**			1.9				2

### Conformational changes in PfTMK domains and loops

**Fig 4:**
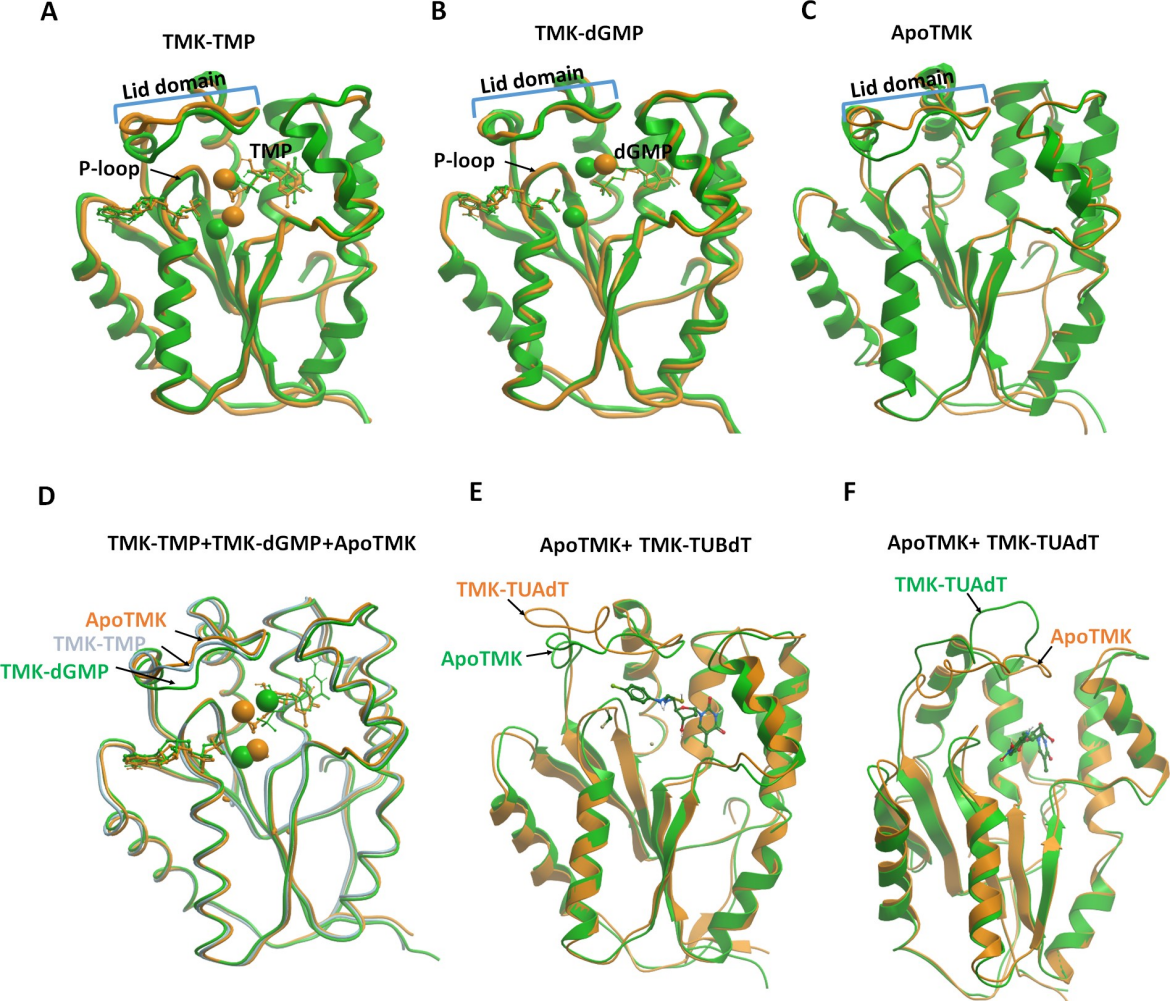
Changes in PfTMK folding and structure after MD. (A) Alignment of TMK-TMP before (green) and after (brown) MD (B) Alignment of TMK-dGMP before (green) and after (brown) MD (C) Alignment of ApoTMK (brown), TMK-TMP (grey) and TMK-dGMP (green) after 50 ns MD (D) Alignment of ApoTMK (green) and TMK-TUBdT (brown) after 50 ns MD (E) Alignment of ApoTMK (green) and TMK-TUAdT (brown) after 50 ns MD. The brown/green spheres represent the bound magnesium ion.

Conformational changes in PfTMK were most observed in the lid domain and in the loops connecting PfTMK`s helices and sheets. Comparison of PfTMK structures before and after MD simulation reveals changes in the lid domain in TMK-TMP (Fig 4A) and ApoTMK (Fig 4C). Such changes were not observed with TMK-dGMP (Fig 4B). A similar finding was observed in PfTMK`s loops. The most important P-loop was more or less stabilized with ApoTMK and TMK-dGMP and showed little changes in RMSDs in TMK-TMP. Fig 4D shows the alignment of ApoTMK, TMK-TMP and TMK-dGMP after the end of 50 ns MD. The lid domain was closer to the P-loop and closing the active site with dGMP followed by TMP and lastly the ApoTMK. In both urea derivatives of thymidine, there was an outward orientation of the lid domain especially with the α-thymidine derivatives (Fig 4E and 4F).

### Ligand interaction profiles

**Fig 5:**
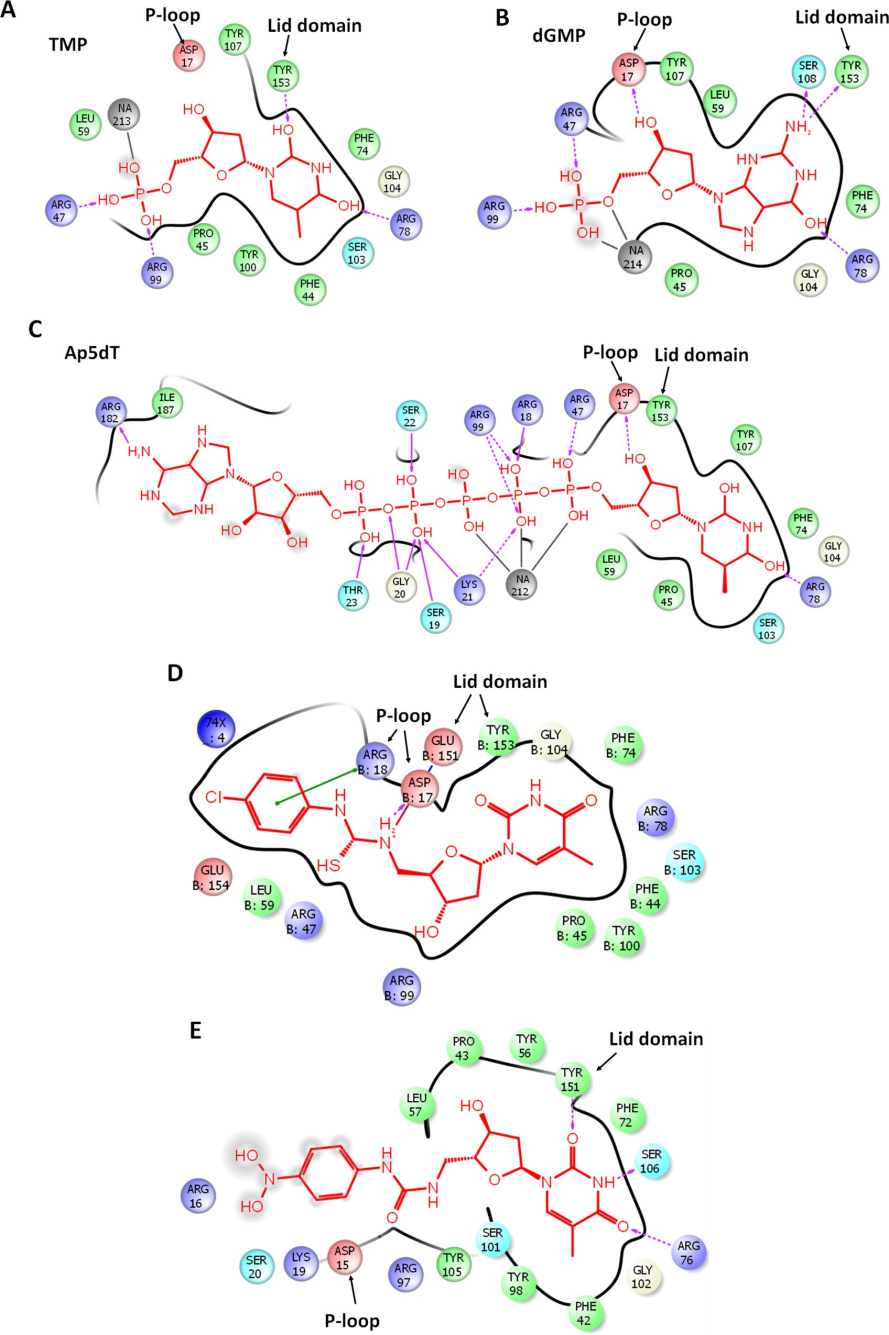
Ligand interactions of substrates and inhibitors with PfTMK. Arrows indicate hydrogen bonding (backbone). Dashed arrows indicate hydrogen bonding (side chain). The direction of the arrow is toward the recipient side.

For further investigation of substrate binding preferences of PfTMK, the ligand interaction profiles were analysed (Fig 5). Both TMP and dGMP retained similar hydrogen bonding profile (Fig 5A and 5B) except for interactions with P-loop, where dGMP interacts with Asp17. All compounds interact with almost one residue in the lid domain, Tyr153 except TUBdT and TUAdT which showed multiple interactions with several residues in the lid domain and P-loop (Fig 5D and 5E).

### Inhibitory studies

Thymidine derivatives were used in most PfTMK assays [2, 6–8, 10]. To gain more insights into the interaction of dG derivatives with PfTMK, a set of compounds were checked for their inhibitory profile (Table 2). dG and 8-Hydroxy-2'-deoxyguanosine showed moderate inhibition, ki = 270 and 180 **μ**M, respectively. The most potent inhibition was by guadecitabine (Ki = 90 **μ**M). Guanosine diphosphate mannose, which is a ribonucleotide derivative, was unable to inhibit PfTMK, implying the importance of dG derivatives as PfTMK inhibitors.

## Discussion

TMK has been introduced as a potential drug target against several microorganisms e.g. *Mycobacterium tuberculosis* [11, 12], *Vaccinia virus* [13] and Gram positive bacteria [14, 15]. PfTMK showed structural features which suggest using it as a target to develop new anti-plasmodial drugs [1, 5, 10]. Yet, further investigations are required to improve the inhibitors efficiency and understand the mechanisms underlying PfTMK recognition of substrates and inhibitors [8, 10]. We used the MD approach to further investigate the structural bases of inhibitors interactions as well as suggesting future optimization experiments. Docking and MD simulation are the gold standards in estimating the recognition of small molecules by protein targets which can help in sampling, confirmation and analysis of proteins complexes [16–18].

### Lid domain is the most reactive substructure by MD

The lid domain showed variable orientations and fluctuations in response to the nature of the bound compound. The higher RMSD in the lid domain of free PfTMK is due to the free movement of loops, beta sheets and helices in the absence of bound substrates. The changes in residues fluctuation weredependent on the type of bound substrate. The most sensitive region with respect to residue fluctuations was the residues of the lid domain. While several other regions of residue fluctuation were observed, this might be due to the trial of PfTMK side chains to reach a stable energy state. The observed decrease in the fluctuation of lid domain residues movement is therefore due to the strength of interaction with the bound compound to PfTMK active site.

### The natural substrates have different PfTMK structural dynamics

TMP is the default substrate for TMKs. The unique feature of PfTMK is its ability to bind dGMP and catalyze its phosphorylation with high efficiency comparable to TMP. Previous studies revealed a common binding site for both TMP and dGMP. In this study, MD revealed different binding and structural dynamic profiles. In comparison with TMP, dGMP showed i) more stable trajectories and lower RMSD during the simulation time ii) lower receptor RMSD iii) lower RMSD of lid domain iv) lid domain is closer to the active site or more firmly closing the active site v) lower changes in P-loop dynamics vi) dGMP interacts with Asp17 in P-loop.

The changes in PfTMK after MD reveal considerable changes at P-loop and lid domain, which are the most important partners in catalytic activity. In order to pack the guanine ring instead of the thymine ring, dGMP interacts with the P-loop through hydrogen bonds and elucidates lower packing of the lid domain toward closing the active site. This conformation is reflected by stable trajectories during MD, lower RMSD values, lower RMSF and generalized structure stability.

PfTMK inhibitory studies indicated that thymidine derivatives have lower ki values compared with guanine derivatives and were more efficiently inhibiting both TMK and dGMP catalytic pathways [1, 2, 6, 7]. This may be due to the observed slight outward orientation of the lid domain with TMP-TMK in this study, which might not be favourable for guanine derivatives by esteeming more closed site conformation.

### Conformational instability with AZTMP

PfTMK can efficiently phosphorylate AZTMP, which is a feature of prokaryotic enzymes but not in eukaryotic TMKs [5]. However, by MD simulation, it is evident that the PfTMK structure is more unstable with large mean backbone and receptor RMSDs. Additionally, there were large flaws in RMSF in residues in one monomer implying asymmetric changes across PfTMK dimer residues.

### The catalytic properties of PfTMK is derived by a unique structural plasticity

The proteins structure plasticity affects their adaptation to perform various catalytic activities and extreme conditions [19, 20]. By MD, PfTMK bears prominent structure plasticity. Despite noticeable distinctions in catalytically important PfTMK substructures e.g. P-loop and lid domain, PfTMK was able to efficiently phosphorylate different substrate families such as TMP, dGMP and AZTMP.

### Hyper branched and urea derivatives of thymidine

5'-urea-α- and β-thymidine derivatives were moderate inhibitors of PfTMK [8]. Ap5dT is a strong inhibitor of thymidylate kinases [21]. It drives its inhibitory potency by showing conserved interactions of thymidine moiety as well as a strong interaction network with the P-loop residues, Glu14-Lys21 (Fig 5C). The obtained lower IC50 of α-thymidine derivatives might be due to its larger size and extension to the phosphorylating site without significant interactions with the P-loop residues (Fig 5D and 5E). After MD simulation, there was a significant reorientation of the P-loop and lid domain in the substituted urea derivatives of thymidine, resulting in the loss of some significant interactions with Gly20 and Lys21.

α-thymidine derivatives formed more stable complexes with PfTMK in comparison with beta derivatives. They showed lower total and receptor RMSD. A constant feature in both TUAdT and TUBdT is the high residue fluctuations at the N-terminal portions of PfTMK that are noticeably higher than other compounds as well as the prominent deviation of the lid domain away from the active site after MD. Both TUAdT and TUBdT were moderate inhibitors of PfTMK [8]. However, TUAdT were more potent against the growth of plasmodium cultures [8]. In the view of the obtained MD profiles, the stronger antimalarial effects of TUAdT are supported by more stable complexes and favourable conformational changes during their interaction with PfTMK.

### dG derivatives

**Fig 6:**
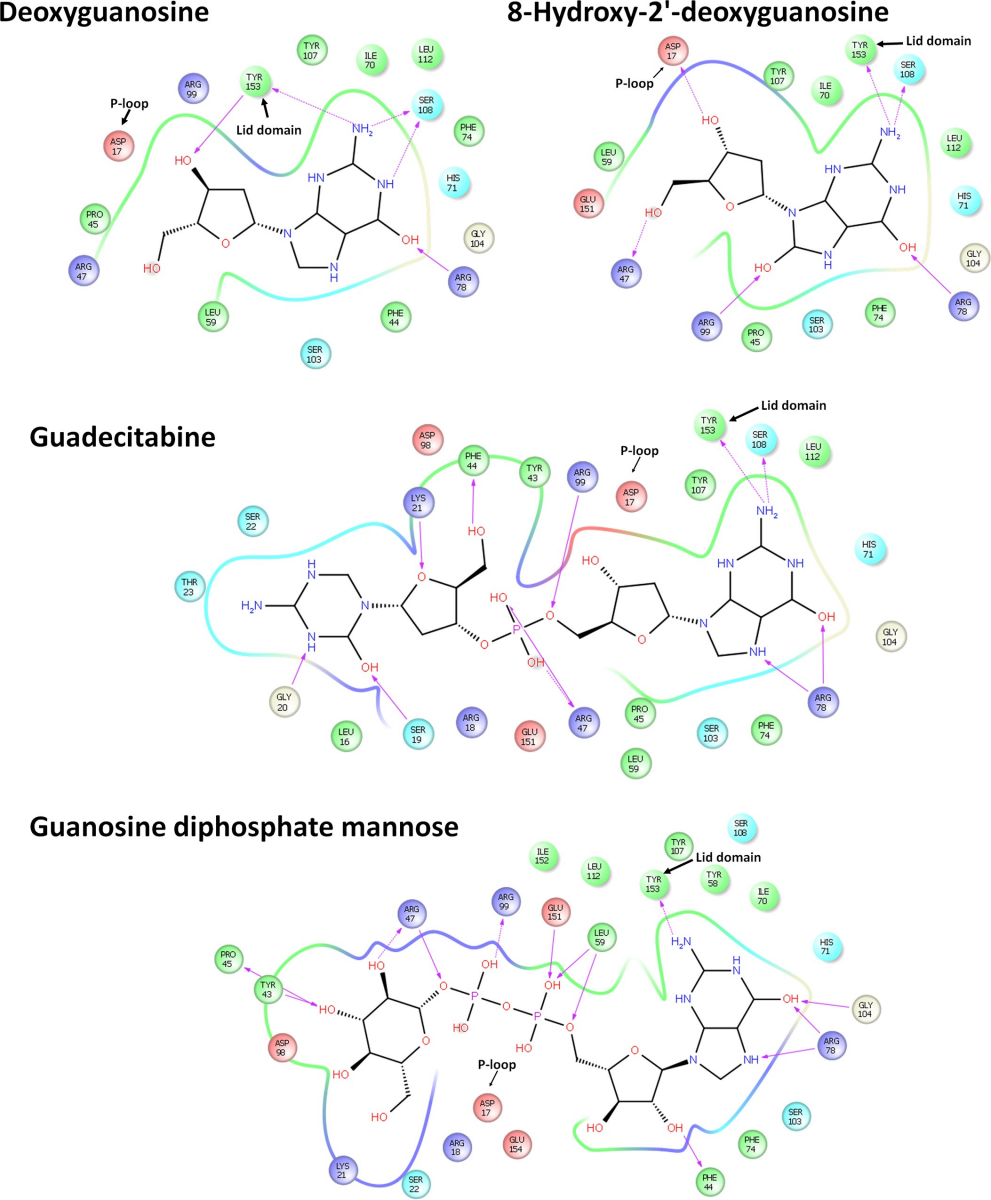
The ligand binding interactions of dG derivatives. Arrows indicate hydrogen bonding (backbone). Dashed arrows indicate hydrogen bonding (side chain). The direction of the arrow is toward the recipient side.

Guanosine diphosphate mannose did not produce any decrease in PfTMK. This confirms the previous finding that ribonucleotides and their derivatives cannot inhibit PfTMK [2]. dG and 8-Hydroxy-2'-deoxyguanosine showed moderate inhibition, which is noticeably improved by guadecitabine. The binding profiles indicate that all compounds were showing conserved interaction with PfTMK including interaction with the P-loop and lid domain (Fig 6). Despite the high docking score and strong network of the interaction of guadecitabine (Fig 6), the observed Ki value is still within a moderate inhibitory range. This might be due to loss of interaction with P-loop Asp17.

Guadecitapine produced the lowest inhibitory potential. Additionally, in docking studies, it showed the highest energy of interaction of -242. The correlation between the estimated experimental and theoretical values suggests the importance of further modifications of this compound to get stronger inhibitors. The purine ring of guadecitabine significantly contributed to the recognition of the compound at the thymidine binding. The 6–OH group makes hydrogen bonds with the side chain of ARG18 (Fig 6). Since stronger hydrogen bonds evolve between the hydroxyl and amino group, this might be an additional factor for the strength of guadecitabine as compared with adenine derivatives. The stronger inhibition produced by guadecitabine as compared with deoxyguanosine and 8-Hydroxy-2'-deoxyguanosine could be attributed to the presence of phosphorus and the extension of its interaction to the phosphorylation as well as ATP binding sites. There was an almost conserved interaction of guadecitabine and Guanosine diphosphate mannose. Additionally, Guanosine diphosphate mannose showed high binding energy. However, the experimentally found weak inhibitory properties could be attributed to the loss of the unfavourable steric interaction between the ribose 3-OH with PHE34, which favours the selection of deoxyribonucleotides over ribonicleotides.

## Conclusions

PfTMK binds several substrate families comprising thymidylate, guanylate and deoxyguanylte. We used MD simulation to discuss the structural bases and changes in PfTMK structure in response to different ligands. Additionally, we used inhibitory studies to evaluate the inhibitory effects of a set of purine compounds. PfTMK has structural plasticity to adapt several substrates and undergo several structural conformation while bearing efficient catalytic power. Interaction with the P-loop and lid domain residues accounts for the major changes associated with structure arrangement and substrate binding. dG derivatives remains less efficient than thymidine derivatives in the inhibition of PfTMK. The binding of PfTMK with purine compounds was associated with stable and lower RMSD change along simulation time, fixation of the lid domain and limited reorientation of the P-loop residues. This might be due to the considerable variations in PFTMK structure, especially in the lid domain, and in the presence of thymidine or guanine derivatives.
